# Educational differences in cancer mortality among women and men: a gender pattern that differs across Europe

**DOI:** 10.1038/sj.bjc.6604274

**Published:** 2008-02-19

**Authors:** G Menvielle, A E Kunst, I Stirbu, B H Strand, C Borrell, E Regidor, A Leclerc, S Esnaola, M Bopp, O Lundberg, B Artnik, G Costa, P Deboosere, P Martikainen, J P Mackenbach

**Affiliations:** 1 Department of Public Health, Erasmus MC, University Medical Center Rotterdam, Rotterdam, The Netherlands; 2 Division of Epidemiology, Norwegian Institute of Public Health, Oslo, Norway; 3 Agència de Salut Pública de Barcelona, Barcelona, Spain; 4 Department of Preventive Medicine and Public Health, Universidad Complutense de Madrid, Madrid, Spain; 5 INSERM U687, Villejuif, France; 6 Research Unit, Department of Health, Basque Government, Vitoria-Gasteiz, Spain; 7 Institute of Social and Preventive Medicine, University of Zurich, Switzerland; 8 CHESS, Stockholm University, Stockholm, Sweden; 9 Department of Public Health, Faculty of Medicine, Ljubljana, Slovenia; 10 Department of Public Health, University of Turin, Turin, Italy; 11 Interface Demography, Centrum voor Sociologie-VUB, Brussels, Belgium; 12 Department of Sociology, University of Helsinki, Helsinki, Finland

**Keywords:** cancer mortality, men, women, Europe, education, cancer site

## Abstract

We used longitudinal mortality data sets for the 1990s to compare socioeconomic inequalities in total cancer mortality between women and men aged 30–74 in 12 different European populations (Madrid, Basque region, Barcelona, Slovenia, Turin, Switzerland, France, Belgium, Denmark, Norway, Sweden, Finland) and to investigate which cancer sites explain the differences found. We measured socioeconomic status using educational level and computed relative indices of inequality (RII). We observed large variations within Europe for educational differences in total cancer mortality among men and women. Three patterns were observed: Denmark, Norway and Sweden (significant RII around 1.3–1.4 among both men and women); France, Switzerland, Belgium and Finland (significant RII around 1.7–1.8 among men and around 1.2 among women); Spanish populations, Slovenia and Turin (significant RII from 1.29 to 1.88 among men; no differences among women except in the Basque region, where RII is significantly lower than 1). Lung, upper aerodigestive tract and breast cancers explained most of the variations between gender and populations in the magnitude of inequalities in total cancer mortality. Given time trends in cancer mortality, the gap in the magnitude of socioeconomic inequalities in cancer mortality between gender and between European populations will probably decrease in the future.

Socioeconomic inequalities are consistently reported for total mortality and for many causes of death (Bucher and Ragland, 1995; Harding, 1995; Borrell *et al*, 1999; Steenland *et al*, 2004), including higher cancer mortality rates among men of lower socioeconomic status (Doornbos and Kromhout, 1990; Davey Smith *et al*, 1991; Faggiano *et al*, 1995, 1997; Mackenbach *et al*, 1999; Borrell *et al*, 2003; Menvielle *et al*, 2005). In contrast among women, many studies did not find important variations by socioeconomic status (Faggiano *et al*, 1997; Michelozzi *et al*, 1999; Borrell *et al*, 2003; Menvielle *et al*, 2005), although higher total cancer mortality rates with higher or lower socioeconomic position are also reported, depending on the country (Faggiano *et al*, 1995; Mackenbach *et al*, 1999). This raises questions to what extent socioeconomic inequalities in total cancer mortality among women really vary between countries, to which an international comparison of educational disparities in cancer mortality could provide some answers. Previous European comparisons focused only on specific cancer sites (Mackenbach *et al*, 2004; Menvielle *et al*, 2007; Strand *et al*, 2007). Such an international comparison would be informative and may help in understanding ways in which cancer inequalities are related to such national factors as alcohol consumption patterns, the smoking epidemic or past social and sanitary developments.

Using recent large longitudinal data set covering many west European populations, we compared educational differences in total cancer mortality between women and men and investigated which cancer sites explained the differences between gender and populations.

## MATERIALS AND METHODS

Longitudinal data from 12 European populations were used (Madrid, the Basque region, Barcelona, Slovenia, Turin, Switzerland, France, Belgium, Denmark, Norway, Sweden and Finland). Most data sets covered the entire national population except France (a representative sample of 1% of the population), the entire regional population (Madrid, the Basque region) or the population from specific urban areas (Barcelona, Turin). Subjects were selected from census and followed up during the 1990s (Table 1). Subpopulations were excluded from three data sets: foreigners in Switzerland, subjects deceased outside Catalonia in Barcelona and foreigners and subjects born in overseas areas in France.

Analyses were performed among subjects aged 30–74 at the time of the census. The follow-up period was shorter for Belgium, Denmark, the Basque region and Madrid, and to obtain comparable ages at death, analyses were conducted on slightly older age groups at baseline (35–79 for Madrid and 30–79 for Belgium and the Basque region). In the case of the Danish data, no information on socioeconomic position was available for subjects aged over 75.

The linkage between census data and mortality registries was achieved for more than 96% of all deceased persons in almost all populations except Madrid (70%), the Basque region (93%) and Barcelona (94.5%). In these populations, however, no variation in this percentage was found according to age, sex or socioeconomic position (in the Basque region unfortunately, this check could not be conducted for socioeconomic status). To avoid underestimation of absolute mortality rates in these three populations, observed absolute mortality was increased using correction factors (1/0.70, 1/0.93 and 1/0.945, respectively).

Socioeconomic status was measured using education declared at the census at the beginning of the follow-up period and classified into three categories, according to the ISCED (International Standard Classification of Education) classification: 0–2 (lower secondary or less), 3–4 (upper secondary), 5–6 (post-secondary). The percentage of missing values was low: 6% for Belgium and less than 3% for other populations; these subjects were excluded from analysis.

Causes of death were obtained by linkage with death registries. Analyses were conducted for total cancer mortality (ICD 9: 140–249), and for the following sites: lung (ICD 9: 162–3, 165), upper aerodigestive tract (UADT: pharynx, oesophagus and larynx) (ICD 9: 140–50, 161), colorectal (ICD 9: 153–4), stomach (ICD 9: 151), leukaemia and Hodgkin's disease (ICD 9: 201, 204–8), kidney and bladder (ICD 9: 188–9), liver (ICD 9: 155), pancreas (ICD 9: 157), breast (ICD 9: 174–5), cervix (ICD 9: 180), prostate (ICD 9: 185), other neoplasms (ICD 9: rest 140–249).

The magnitude of socioeconomic inequalities in mortality was estimated in both absolute and relative terms. For the latter, we computed relative indices of inequality (RII) using Poisson regression, calculating RII as a ranked variable, which, for each educational group, specifies the mean proportion of the population with a higher level of education, for example that of the lowest group is calculated as the proportion of the population with middle or high education, plus half of the proportion of the population with a lowest level. Relative indices of inequality are then computed by regressing the mortality on this ranked variable. Thus, RII express inequality within the whole socioeconomic continuum and can be interpreted as the ratio of mortality rates between the two extremes of the educational hierarchy. As it takes into account the size and relative position of each group, it is well adapted to compare populations with different educational distributions (Pamuk, 1985; Mackenbach and Kunst, 1997). Analyses were conducted for each population separately, and to assess whether differences were significant, we tested the interaction between country and education in a model that included all populations.

To estimate absolute socioeconomic inequalities, we computed absolute mortality rate differences between the lowest and the highest educational level, both for all cancer mortality and for specific types. Age-standardised mortality rates were computed, using the population of EU-15 plus Norway of 1995 as the standard.

## RESULTS

Large differences were observed in educational distribution between countries and among men and women (Table 1). The percentage of subjects with post-secondary education was lower among women in all populations, except Sweden, and was below 10% among women in Turin, France and Slovenia.

**Figure 1:**
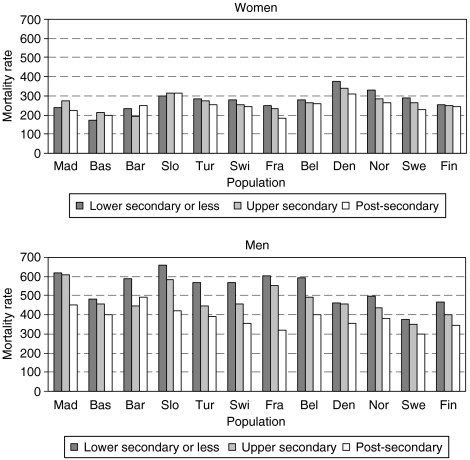
Total cancer mortality rates (per 100 000 person years) by education among women and men, by population. Age-adjusted mortality rates using direct standardisation.

Figure 1 presents total cancer mortality by educational level. Rates were markedly lower among women, and among those with the lowest education were lower than among men with the highest education in all populations except Denmark. Total cancer mortality rates were generally higher among men with lower educational levels. Among women, however, the gradient was much narrower and was not observed in the Spanish regions, Slovenia and Finland. The lowest total cancer mortality rates were observed in the Spanish regions and in France for women, in the Nordic countries, especially Sweden, for men (Tables 2, 3). Breast, lung and colorectal cancers accounted for 39–46% of all cancers among women except in Denmark (52%). Among men, prostate, lung and colorectal cancers accounted for 47–52% of all cancers, except in France (42%), the Basque region (44%) and Belgium (57%).

The RII for total cancer mortality by population are presented in Table 2 (women) and Table 3 (men). We can distinguish three main patterns: in Denmark, Norway and Sweden, we observed higher rates among less-educated men and women, with significant RII around 1.3–1.4; in France, Switzerland, Belgium and Finland, RII were significantly higher than 1 among men and women, but larger among men (around 1.7–1.8) than among women (around 1.2); in Madrid, Barcelona, Slovenia and Turin, RII were significant among men (from 1.29 to 1.88) but not among women (borderline in Turin); among women, it was even significantly lower than 1 in the Basque region.

For some cancer sites among women, inequalities in RII did not significantly differ between populations (Table 2). No educational differences were found for leukaemia and Hodgkin's disease. Small inequalities favouring high-educated people were found for colorectal cancer, larger for liver cancer and especially for cervical and stomach cancers. However, analyses for cervix cancer were based on small number of deaths. For breast cancer, RII were significantly lower than 1 in all populations, except in Turin, France and Switzerland.

A clear north–south gradient was found for lung cancer with RII significantly lower than 1 in the three Spanish regions and Slovenia but significantly higher than 1 in Switzerland, Belgium, and the Nordic countries. No significant association between education and lung cancer mortality was observed in France and Turin. Statistically significantly higher UADT cancer mortality rates were found among less-educated women in Switzerland, France and the Nordic countries, even though mortality rates were low. Contrasting situations were found for pancreatic and kidney and bladder cancer. Relative indices of inequality lower than 1 were found in Spanish populations and in Slovenia, whereas the highest RII were observed in the Nordic countries. For the category ‘other cancers’, the inequalities were remarkably similar, ranging from 1.17 to 1.30 in 10 out of 12 countries.

Relative indices of inequality among men by population and cancer site are presented in Table 3. No significant interaction between education and populations was observed for leukaemia and Hodgkin's disease, prostate and pancreatic cancer. Mortality did not differ by educational level for leukaemia and Hodgkin's disease and prostate, and we found slightly higher pancreatic cancer mortality among less-educated people.

Large variations between populations were found for lung, UADT and stomach cancers even though higher mortality rates among less-educated men were found in all populations. Larger RII were found in northern countries and Switzerland for lung cancer, in Slovenia, Switzerland and France for UADT cancers and in the Southern populations for stomach cancer. A contrasting picture was found for liver cancer with no educational differences in mortality in some populations (Belgium, Norway, Slovenia, Basque region) and higher rates among less-educated people in others. Higher rates for less-educated men were found for colorectal cancer and slightly higher for bladder and kidney cancers (RII around 1.5).

Absolute mortality rate differences by site are presented in Table 4. With a few sites (lung, UADT and breast) explaining most of these by country and gender. Colorectal and prostate cancers do not explain much of the differences in absolute socioeconomic inequalities. Among women, the populations with larger excess deaths are same for both less-educated women with lung cancer and more-educated women with breast cancer (Norway, Sweden and Denmark). These populations contrast with populations with excess deaths among more-educated women with both breast and lung cancer (Spanish populations and Slovenia).

## DISCUSSION

Educational differences in total cancer mortality were equally high among women and men in Norway, Sweden and Denmark, but inequalities among women were smaller as compared with men, non-existent in Madrid, Barcelona, Turin and Slovenia, and even the reverse in the Basque region. Variations in inequalities in mortality from lung, UADT and breast cancers explained most of the differences between men and women and within European populations.

In France and Switzerland, foreigners were excluded. Migrants have lower cancer mortality for most sites, except some sites specific to their native country (nasopharynx, gallbladder or liver (because of exposure to hepatitis B virus)) (Bouchardy *et al*, 1994, 1995, 1996). As foreigners have generally lower educational levels, their exclusion may have led to some overestimation of inequalities in cancer mortality in France and Switzerland.

Potential differences in national practices in causes of death coding are relevant, all our data came from populations where this was reliable. Our results would be biased only if diagnosing practices differed by socioeconomic status of the deceased. If no supporting evidence exists, this cannot be entirely excluded.

Part of the large variation between populations in educational distribution may be due to real differences. Nevertheless, despite our common classification for all countries, national educational categories may be applied to this in different ways. We evaluated the sensitivity of the results to alternative classifications, including a classification comprised of four educational levels, by distinguishing between subjects who completed lower secondary from those with primary education only. In addition, we applied another classification in which distributions were as similar as possible between populations. The results were quite robust to these alternatives with only slight changes in RII and the relative order unaltered, as we infer that any misclassification is unlikely to have biased the main results.

As indicated in Introduction, other studies also observed that more-educated men have lower cancer mortality. Studies among women were fragmentary, mainly for southern Europe and did not consistently report socioeconomic inequalities. We found a north–south gradient among women with no inequalities, in the south and large inequalities in the Nordic countries.

Our results agree with previous reports on higher cervix cancer mortality with lower education, and no association between education and mortality for leukaemia and Hodgkin's disease or not clearly for pancreatic, kidney and bladder cancers (Davey Smith *et al*, 1991; Faggiano *et al*, 1995; van Loon *et al*, 1995; Faggiano *et al*, 1997; Fernandez and Borrell, 1999; Michelozzi *et al*, 1999). However, from our European overview, kidney and bladder cancers have consistently higher mortality among less-educated men. Previous studies found conflicting results for colon and rectal cancer (Davey Smith *et al*, 1991; Faggiano *et al*, 1995, 1997; Fernandez and Borrell, 1999). Similarly, to results found in two US studies in the 1980s (Singh *et al*, 2002; Steenland *et al*, 2002), we found higher mortality from colorectal cancer among lower socioeconomic groups in European populations in the 1990s, both among men and women.

We observed large disparities between populations in the gender difference in the magnitude of socioeconomic inequalities in total cancer mortality, being much smaller in Sweden, Norway and Denmark. Compared with other countries, inequalities in these countries are higher among women and smaller among men. Situation by site can explain these differences.

Patterns of educational differences in lung cancer mortality differ strongly by population and gender as in another work (Mackenbach *et al*, 2004), and is now confirmed across a broader set of populations, including France, Slovenia and the Basque region. This reflects differences in the diffusion of the smoking epidemic (Lopez *et al*, 1994), in which higher smoking rates are first observed among subjects of higher socioeconomic status. The smoking epidemic was less advanced in Spain and Slovenia, where we found higher smoking prevalence among women with high education but already reverse patterns among men. In contrast, this epidemic was in its final stage in Nordic countries, Belgium and Switzerland, with higher tobacco consumption among women and men with low education. France and Italy showed an intermediate situation with no clear association between education and smoking prevalence among women (Giskes *et al*, 2005; Huisman *et al*, 2005). Male–female smoking differences by education are less pronounced in Nordic countries or Belgium than in more southern countries, reflecting the clear north–south contrast observed among women for socioeconomic inequalities in smoking. Thus, the gradual spread of the smoking epidemic not only underlies international variations in socioeconomic inequalities in lung cancer mortality, but also explains why southern populations still show much larger inequalities among men than women.

Alcohol consumption is likely to be involved in liver and UADT cancers. Socioeconomic inequalities were also observed in most populations among women. However, because of low overall mortality rates, UADT cancers hardly contribute to these inequalities among women. We found especially large socioeconomic inequalities favouring high-educated men for these cancers in France and the Spanish populations, as described elsewhere (Menvielle *et al*, 2007). Data on socioeconomic inequalities in alcohol consumption are still fragmentary, but differences between countries are suggested, especially among men (Cavelaars *et al*, 1997; Kunst AE and Schaap MM, personal communication). A north–south contrast was found among men. In Italy and Spain, alcohol consumption was higher among less-educated men, whereas in more northern countries, alcohol consumption was higher among more-educated men. Among regular drinkers, average alcohol consumption levels were strongly associated with low education among men in Spain and Italy.

Higher breast cancer mortality is generally observed among more-educated women, mainly due to differences in reproductive behaviour, and especially delayed first birth (Braaten *et al*, 2004; Strand *et al*, 2005). We found variations among populations in socioeconomic inequalities, although their relative magnitude did not strongly differ among them; the magnitude of absolute inequalities nevertheless substantially differed because of variations in national breast cancer mortality rates, which affected total cancer mortality among women. In Norway, Sweden and Denmark the ‘moderating’ effect of breast cancer on inequalities in total cancer mortality is less strong than in Madrid or Slovenia.

Similar to others, we found higher stomach cancer mortality among the less educated (Davey Smith *et al*, 1991; Faggiano *et al*, 1995, 1997; Fernandez and Borrell, 1999; Michelozzi *et al*, 1999; Menvielle *et al*, 2005). Among such cancers, stomach cancer is the only one for which inequalities tend to be smaller in the Nordic countries. As *Helicobacter pylori* infection at young age is a risk factor for stomach cancer, this is likely to be associated with factors linked to childhood living conditions (Boffetta, 1997), the smaller inequalities in Nordic countries might be due to better social and sanitary conditions in the past. Although there is no direct evidence for this, relevant factors may include the active housing policies as well as the more egalitarian social and economic policies developed in Nordic countries since the early 20th century.

Differences between countries in health care access probably also explain part of our findings, especially for cancers with high survival. Nevertheless, we did not observe socioeconomic inequalities for leukaemia, Hodgkin's disease or prostate cancer mortality. As these cancers lack socioeconomic inequalities in incidence and have a good prognosis, this result suggests that the contribution of medical care to inequalities in cancer mortality in western Europe is modest.

The large geographical and gender differences of socioeconomic inequalities in cancer mortality suggest a large potential for change. Recent studies have suggested smaller educational differences in breast cancer mortality among younger women in many European populations (Martikainen and Valkonen, 2000; Menvielle *et al*, 2006; Strand *et al*, 2007). On the other hand, as southern European countries have still to experience the last stage of the smoking epidemic, higher lung cancer mortality rates among less-educated women may result there as well. Given these trends, variations between countries and genders in socioeconomic inequalities in cancer mortality are likely to reduce. In any case, future trends in socioeconomic inequalities in cancer mortality will be largely due to trends in lung, UADT and breast cancers. Consequently, tobacco control, alcohol policies and breast cancer screening should ensure that they reach lower, as well as higher, socioeconomic groups.

## Figures and Tables

**Table 1:** Socio-demographic characteristics, by gender and population

		**Women**				**Men**			
				**Education (%)**				**Education (%)**	
**Population**	**Follow-up**	**Person years**	**Cancer deaths**	**Middle/high**	**High**	**Person years**	**Cancer deaths**	**Middle/high**	**High**
Madrid	May 1996–Dec 1997	2 030 998	3331	24.2	11.7	1 756 059	6133	35.9	18.5
Basque region	May 1996–Jun 2001	3 186 595	5431	25.4	12.0	2 985 865	11 737	34.4	14.3
Barcelona	Jan 1992–Dec 2001	4 489 610	11 450	23.1	13.8	3 714 380	20 253	34.8	19.5
Slovenia	Apr 1991–Dec 2000	5 158 738	14 316	43.5	9.1	4 614 864	20 105	62.6	12.4
Turin	Nov 1991–Oct 2001	2 611 141	7837	24.5	6.8	2 611 968	11 294	32.8	10.6
Switzerland	Dec 1990–Dec 2000	15 113 931	39 612	60.3	7.2	12 969 989	53 679	80.4	24.3
France	Mar 1990–Dec 1999	1 270 981	2883	37.2	10.0	1 135 299	5375	49.4	12.7
Belgium	Mar 1991–Dec 1995	13 688 568	37 354	32.7	13.9	12 700 788	58 760	38.7	16.8
Denmark	Jan-1996–Dec-2000	7 033 258	24 170	49.9	19.9	6 893 032	25 915	59.9	20.1
Norway	Nov 1990–Nov 2000	10 424 746	69 894	63.8	16.8	10 021 675	38 722	70.1	21.7
Sweden	Jan 1991–Dec 2000	22 116 058	61 446	59.1	19.0	21 421 623	70 339	59.7	16.4
Finland	Dec 1990–Dec 2000	13 478 149	32 880	48.6	19.7	12 396 052	39 734	51.2	21.5

Middle/high: upper secondary education or more, high: post-secondary education.

**Table 2:** Mortality rates^a^ and relative indices of inequality related to education by cancer site among women, by population

	**All cancers**		**Lung**		**UADT**		**Leukaemia and Hodgkin's disease**		**Breast**		**Cervix**	
	**MR**	**RII (95% CI)**	**MR**	**RII (95% CI)**	**MR**	**RII (95% CI)**	**MR**	**RII (95% CI)**	**MR**	**RII (95% CI)**	**MR**	**RII (95% CI)**
Madrid	242	0.89 (0.74–1.08)	15	0.34 (0.17–0.65)	5	1.02 (0.27–3.79)	8	0.94 (0.34–2.62)	48	0.51 (0.35–0.74)	5	4.87 (0.92–25.90)
Basque region	178	0.63 (0.54–0.75)	13	0.29 (0.17–0.48)	4	0.76 (0.26–2.25)	5	0.73 (0.28–1.92)	36	0.61 (0.43–0.86)	3	4.31 (0.79–23.49)
Barcelona	246	1.04 (0.94–1.16)	16	0.49 (0.34–0.69)	5	0.79 (0.40–1.56)	8	1.20 (0.66–2.19)	51	0.79 (0.64–0.97)	5	1.43 (0.71–2.91)
Slovenia	302	0.93 (0.87–1.00)	29	0.49 (0.40–0.61)	5	1.56 (0.89–2.74)	8	0.73 (0.48–1.10)	57	0.69 (0.59–0.80)	9	2.10 (1.39–3.17)
Turin	284	1.12 (0.98–1.27)	32	0.74 (0.52–1.06)	6	1.16 (0.49–2.77)	10	0.95 (0.49–1.82)	61	0.93 (0.72–1.20)	3	7.77 (1.48–40.86)
Switzerland	263	1.21 (1.16–1.26)	29	1.35 (1.20–1.52)	7	1.28 (1.00–1.63)	8	1.05 (0.84–1.31)	59	0.96 (0.88–1.04)	5	1.78 (1.33–2.38)
France	239	1.30 (1.08–1.57)	18	0.84 (0.45–1.55)	7	4.41 (1.38–14.08)	8	0.87 (0.31–2.48)	54	1.19 (0.82–1.71)	4	3.05 (0.65–14.45)
Belgium	276	1.17 (1.11–1.23)	26	1.61 (1.35–1.93)	7	0.88 (0.64–1.20)	9	1.10 (0.82–1.47)	66	0.83 (0.75–0.91)	5	2.36 (1.58–3.51)
Denmark	356	1.33 (1.26–1.40)	80	2.31 (2.05–2.60)	11	1.60 (1.17–2.19)	8	1.09 (0.77–1.55)	67	0.83 (0.74–0.93)	9	2.26 (1.61–3.16)
Norway	303	1.38 (1.32–1.44)	43	2.77 (2.45–3.14)	6	1.94 (1.40–2.69)	7	0.89 (0.66–1.19)	51	0.78 (0.70–0.86)	9	3.28 (2.52–4.27)
Sweden	271	1.31 (1.27–1.35)	37	1.79 (1.63–1.96)	5	1.70 (1.33–2.17)	8	0.98 (0.81–1.18)	45	0.88 (0.82–0.95)	5	2.59 (2.02–3.32)
Finland	257	1.20 (1.15–1.26)	24	1.85 (1.56–2.21)	6	2.00 (1.41–2.82)	9	1.10 (0.84–1.44)	46	0.85 (0.76–0.94)	4	3.47 (2.20–5.46)
Interaction^b^		<0.005		<0.005		<0.005		NS		NS		NS
												
	**Stomach**		**Liver**		**Kidney and bladder**		**Pancreas**		**Colorectum**		**Other**	
	**MR**	**RII (95% CI)**	**MR**	**RII (95% CI)**	**MR**	**RII (95% CI)**	**MR**	**RII (95% CI)**	**MR**	**RII (95% CI)**	**MR**	**RII (95% CI)**
Madrid	17	2.41 (0.97–5.97)	12	0.68 (0.27–1.68)	6	0.64 (0.18–2.26)	12	0.73 (0.31–1.74)	32	1.19 (0.67–2.10)	83	1.29 (0.91–1.83)
Basque region	11	1.80 (0.76–4.24)	7	1.29 (0.43–3.89)	6	0.54 (0.21–1.40)	10	0.38 (0.19–0.75)	20	0.77 (0.44–1.33)	62	0.63 (0.47–0.85)
Barcelona	11	2.02 (1.17–3.50)	12	1.63 (0.97–2.76)	6	1.26 (0.64–2.47)	11	0.67 (0.43–1.05)	32	1.36 (1.00–1.84)	74	1.30 (1.07–1.57)
Slovenia	24	1.51 (1.15–1.99)	7	1.55 (0.93–2.59)	9	0.64 (0.42–0.96)	16	0.86 (0.63–1.18)	38	1.28 (1.03–1.59)	100	1.04 (0.92–1.18)
Turin	13	3.16 (1.48–6.71)	10	2.77 (1.16–6.59)	8	2.52 (0.94–6.75)	15	1.25 (0.69–2.24)	34	0.97 (0.67–1.41)	93	1.17 (0.94–1.47)
Switzerland	9	2.34 (1.86–2.95)	5	1.49 (1.10–2.03)	11	1.89 (1.54–2.32)	16	1.27 (1.08–1.50)	26	1.02 (0.90–1.16)	89	1.23 (1.15–1.32)
France	8	4.95 (1.24–19.68)	6	1.59 (0.45–5.63)	8	0.96 (0.35–2.64)	12	1.86 (0.73–4.70)	27	1.24 (0.70–2.17)	86	1.26 (0.93–1.72)
Belgium	10	2.44 (1.74–3.41)	5	1.38 (0.90–2.11)	11	1.68 (1.24–2.28)	13	1.31 (1.01–1.70)	34	1.24 (1.05–1.45)	90	1.22 (1.11–1.34)
Denmark	7	1.51 (1.01–2.25)	5	1.72 (1.10–2.70)	14	1.80 (1.35–2.40)	17	0.94 (0.74–1.19)	40	1.18 (1.01–1.39)	100	1.23 (1.12–1.36)
Norway	12	1.77 (1.41–2.20)	2	1.32 (0.79–2.18)	12	2.09 (1.66–2.62)	18	1.39 (1.16–1.67)	42	1.25 (1.11–1.41)	100	1.26 (1.17–1.36)
Sweden	9	1.83 (1.53–2.20)	7	1.80 (1.45–2.24)	12	1.71 (1.46–2.01)	19	1.23 (1.09–1.39)	29	1.31 (1.18–1.45)	96	1.29 (1.22–1.37)
Finland	14	1.39 (1.12–1.73)	7	1.81 (1.31–2.50)	10	1.10 (0.85–1.41)	20	1.45 (1.20–1.75)	24	1.03 (0.88–1.21)	93	1.21 (1.12–1.32)
Interaction^b^		NS		NS		<0.005		<0.005		NS		<0.005

MR=mortality rates; RII=relative indices of inequality; UADT=upper aerodigestive tract.

a Age-adjusted mortality rates using direct standardisation, per 100 000 person years.

b *P*-value for interaction test between education and population.

**Table 3:** Mortality rates^a^ and relative indices of inequality related to education by cancer site among men, by population

	**All cancers**		**Lung**		**UADT**		**Leukaemia and Hodgkin's disease**		**Prostate**			
	**MR**	**RII (95% CI)**	**MR**	**RII (95% CI)**	**MR**	**RII (95% CI)**	**MR**	**RII (95% CI)**	**MR**	**RII (95% CI)**		
Madrid	593	1.52 (1.34–1.72)	175	1.53 (1.22–1.92)	56	2.58 (1.71–3.89)	16	1.75 (0.84–3.65)	38	1.12 (0.67–1.87)		
Basque region	473	1.29 (1.17–1.43)	125	1.31 (1.08–1.59)	59	2.04 (1.53–2.71)	11	0.90 (0.48–1.71)	29	0.94 (0.61–1.45)		
Barcelona	586	1.57 (1.47–1.68)	169	1.80 (1.60–2.04)	52	3.12 (2.48–3.91)	15	1.04 (0.71–1.52)	34	1.01 (0.77–1.32)		
Slovenia	595	1.72 (1.63–1.82)	181	2.08 (1.89–2.29)	62	5.56 (4.70–6.57)	14	1.22 (0.86–1.72)	44	1.18 (0.95–1.47)		
Turin	532	1.88 (1.71–2.06)	179	2.53 (2.13–2.99)	33	3.61 (2.41–5.42)	14	1.07 (0.63–1.80)	31	1.01 (0.69–1.49)		
Switzerland	465	1.83 (1.77–1.89)	127	2.91 (2.73–3.10)	37	4.05 (3.60–4.55)	14	1.19 (0.98–1.43)	56	1.13 (1.03–1.25)		
France	555	1.89 (1.69–2.13)	147	1.64 (1.32–2.03)	78	4.30 (3.10–5.95)	13	1.36 (0.63–2.91)	37	1.04 (0.65–1.65)		
Belgium	555	1.80 (1.73–1.88)	216	3.10 (2.89–3.32)	38	1.74 (1.51–2.00)	16	1.03 (0.83–1.28)	50	1.17 (1.01–1.34)		
Denmark	441	1.31 (1.25–1.37)	128	1.76 (1.61–1.93)	36	1.77 (1.51–2.08)	13	1.02 (0.78–1.32)	43	0.94 (0.81–1.10)		
Norway	449	1.45 (1.39–1.50)	107	2.45 (2.26–2.65)	21	2.27 (1.90–2.71)	11	0.82 (0.65–1.05)	67	0.94 (0.85–1.03)		
Sweden	356	1.32 (1.28–1.35)	70	1.81 (1.69–1.93)	17	2.03 (1.77–2.33)	12	1.12 (0.96–1.32)	59	1.04 (0.97–1.12)		
Finland	437	1.72 (1.64–1.80)	138	3.48 (3.18–3.81)	19	2.38 (1.94–2.94)	13	1.18 (0.93–1.51)	52	1.01 (0.88–1.16)		
Interaction^b^		<0.005		<0.005		<0.005		NS		NS		
												
	**Stomach**		**Liver**		**Kidney and bladder**		**Pancreas**		**Colorectum**		**Other**	
	**MR**	**RII (95% CI)**	**MR**	**RII (95% CI)**	**MR**	**RII (95% CI)**	**MR**	**RII (95% CI)**	**MR**	**RII (95% CI)**	**MR**	**RII (95% CI)**
Madrid	40	2.41 (1.46–3.97)	38	2.76 (1.61–4.74)	41	1.23 (0.77–1.98)	21	0.85 (0.47–1.55)	63	1.08 (0.75–1.56)	105	1.29 (0.97–1.70)
Basque region	36	3.15 (2.03–4.90)	22	1.16 (0.72–1.87)	33	1.62 (1.07–2.45)	18	0.78 (0.49–1.25)	52	0.92 (0.69–1.24)	87	1.02 (0.82–1.28)
Barcelona	29	4.14 (2.95–5.79)	37	1.56 (1.20–2.02)	40	1.31 (1.02–1.67)	26	1.02 (0.75–1.39)	61	1.15 (0.95–1.40)	95	1.22 (1.05–1.43)
Slovenia	59	2.31 (1.93–2.76)	17	1.15 (0.84–1.57)	31	0.95 (0.75–1.20)	24	1.31 (1.00–1.70)	72	0.97 (0.83–1.14)	90	1.10 (0.96–1.26)
Turin	29	4.53 (2.82–7.28)	36	2.49 (1.69–3.68)	39	1.58 (1.11–2.25)	23	0.83 (0.55–1.23)	53	1.46 (1.09–1.95)	95	1.33 (1.08–1.64)
Switzerland	21	2.53 (2.16–2.95)	18	1.68 (1.42–1.98)	30	1.41 (1.24–1.60)	23	1.27 (1.10–1.47)	47	1.27 (1.14–1.40)	93	1.35 (1.26–1.45)
France	22	2.62 (1.39–4.94)	36	2.59 (1.63–4.12)	33	1.33 (0.83–2.14)	24	1.01 (0.60–1.70)	47	1.58 (1.06–2.36)	118	1.73 (1.36–2.20)
Belgium	25	3.19 (2.57–3.96)	12	1.03 (0.79–1.34)	35	1.37 (1.17–1.61)	22	0.97 (0.80–1.17)	52	1.18 (1.04–1.34)	90	1.25 (1.14–1.37)
Denmark	13	2.05 (1.55–2.69)	9	1.39 (1.01–1.93)	34	1.09 (0.92–1.30)	21	1.06 (0.86–1.30)	54	1.03 (0.90–1.18)	90	1.11 (1.00–1.22)
Norway	27	1.89 (1.61–2.21)	4	1.00 (0.68–1.46)	32	1.62 (1.40–1.88)	23	1.35 (1.14–1.60)	61	1.08 (0.97–1.19)	96	1.19 (1.10–1.29)
Sweden	19	1.83 (1.60–2.08)	11	1.68 (1.42–1.98)	26	1.28 (1.15–1.43)	23	1.16 (1.04–1.30)	39	1.11 (1.01–1.21)	81	1.14 (1.07–1.21)
Finland	29	2.36 (1.97–2.83)	13	1.35 (1.05–1.73)	27	1.50 (1.26–1.79)	27	1.16 (0.98–1.37)	36	0.94 (0.81–1.09)	82	1.25 (1.14–1.38)
Interaction^b^		<0.005		<0.005		0.01–0.005		NS		0.025–0.01		<0.005

MR=mortality rates; RII=relative indices of inequality; UADT=upper aerodigestive tract.

a Age-adjusted mortality rates using direct standardisation, per 100 000 person years.

b *P*-value for interaction test between education and population.

**Table 4:** Mortality rate^a^ difference between subjects with lower secondary education or less and subjects with post-secondary education for total cancer mortality and by cancer site, per gender and population

		**Cancer site**											
**Population**	**Total cancer**	**Lung**	**UADT**	**Breast**	**Cervix**	**Prostate**	**Leukaemia and Hodgkin's disease**	**Stomach**	**Liver**	**Bladder and kidney**	**Pancreas**	**Colorectum**	**Other**
*Women*													
Madrid	16	−5	1	−23	4	—	3	4	−1	0	−2	9	27
Basque region	−27	−13	−1	−7	2	—	−4	6	1	1	−1	−5	−6
Barcelona	−18	−8	0	−13	1	—	1	1	1	0	−4	3	0
Slovenia	−16	−18	2	−22	6	—	0	8	2	−1	−3	4	7
Turin	34	−7	0	5	3	—	2	6	6	7	−2	6	7
Switzerland	4	2	0	0	0	—	0	0	0	0	0	0	1
France	65	6	5	2	3	—	4	8	1	2	2	−5	39
Belgium	20	7	−1	−9	3	—	−1	4	1	4	2	3	7
Denmark	67	42	4	−9	6	—	0	2	2	6	1	3	10
Norway	68	31	3	−13	8	—	−1	3	0	6	4	7	18
Sweden	58	18	3	−6	4	—	0	4	3	4	3	5	20
Finland	22	7	3	−10	3	—	0	4	3	0	3	0	9
													
*Men*													
Madrid	165	48	32	—	—	5	4	17	25	8	−2	5	22
Basque region	83	26	19	—	—	−2	−5	18	3	13	4	2	4
Barcelona	102	46	26	—	—	−1	−1	17	10	2	−3	0	6
Slovenia	240	107	74	—	—	5	2	36	2	2	1	7	4
Turin	176	84	14	—	—	−1	0	22	15	16	−2	10	17
Switzerland	214	104	38	—	—	5	2	15	7	9	4	8	22
France	281	83	78	—	—	4	−1	12	25	18	13	13	35
Belgium	188	126	11	—	—	7	1	14	0	8	0	9	12
Denmark	105	63	16	—	—	−1	−1	7	2	7	1	4	8
Norway	119	67	13	—	—	−3	−2	11	1	11	6	4	13
Sweden	77	32	8	—	—	1	1	9	5	6	3	4	8
Finland	123	85	10	—	—	−1	1	13	1	5	1	−2	11

UADT=upper aerodigestive tract.

a Age-adjusted mortality rates using direct standardisation, per 100 000 person years.
